# Effectiveness of active middle ear implant placement methods in pathological conditions: basilar membrane vibration simulation

**DOI:** 10.3389/fneur.2024.1417711

**Published:** 2024-08-08

**Authors:** Sinyoung Lee, Masaomi Motegi, Takuji Koike

**Affiliations:** ^1^Department of Mechanical Engineering, Faculty of Engineering, Graduate Faculty of Interdisciplinary Research, University of Yamanashi, Yamanashi, Japan; ^2^Department of Otolaryngology-Head and Neck Surgery, Gunma University Graduate School of Medicine, Maebashi, Gunma, Japan; ^3^Department of Mechanical and Intelligent Systems Engineering, Graduate School of Informatics and Engineering, The University of Electro-Communications, Tokyo, Japan

**Keywords:** active middle ear implant, finite element method, human cochlear model, floating mass transducer, oval window vibroplasty, round window vibroplasty, mixed hearing loss, otosclerosis

## Abstract

Active middle ear implants (AMEI) amplify mechanical vibrations in the middle ear and transmit them to the cochlea. The AMEI includes a floating mass transducer (FMT) that can be placed using two different surgical approaches: “oval window (OW) vibroplasty” and “round window (RW) vibroplasty.” The OW and RW are windows located on the cochlea. Normally, sound stimulus is transmitted from the middle ear to cochlea via the OW. RW vibroplasty has been suggested as an alternative method due to the difficulty of applying OW vibroplasty in patients with ossicle dysfunction. Several reports compare the advantages of each approach through pre and postoperative hearing tests. However, quantitatively assessing the treatment effect is challenging due to individual differences in pathologies. This study investigates the vibration transmission efficiency of each surgical approach using a finite-element model of the human cochlea. Vibration of the basilar membrane (BM) of the cochlea is simulated by applying the stimulus through the OW or RW. Pathological conditions, such as impaired stapes mobility, are simulated by increasing the stiffness of the stapedial annular ligament. RW closure due to chronic middle ear diseases is a common clinical occurrence and is simulated by increasing the stiffness of the RW membrane in the model. The results show that the vibration amplitude of the BM is larger when the stimulus is applied to the RW compared to the OW, except for cases of RW membrane ossification. The difference in these amplitudes is particularly significant when stapedial mobility is limited. These results suggest that RW vibroplasty would be advantageous, especially in cases of accompanying stapedial mobility impairment. Additionally, it is suggested that transitioning to OW vibroplasty could still ensure a sufficient level of vibratory transmission efficiency when placing the FMT on the RW membrane is difficult due to anatomical problems in the tympanic cavity or confirmed severe pathological conditions around the RW.

## 1 Introduction

Active middle ear implants (AMEI) are used to address conductive or mixed hearing loss by bypassing middle ear issues through mechanical vibrations delivered by an actuator. The Vibrant Soundbridge^®^ (VSB) (MED-EL, Innsbruck, Austria), originally designed for sensorineural hearing loss, was initially developed to transmit sound via the oval window (OW), attaching the floating mass transducer (FMT) to the intact ossicle. Since 2006, alternative applications of the FMT, specifically to the round window (RW), have been explored (1). RW placement facilitates direct vibratory energy transfer to the cochlea, making it a viable option when ossicular reconstruction is not feasible or has failed, and conventional hearing aids are ineffective. Consequently, RW stimulation has become increasingly significant in clinical practice. Several surgeons have reported favorable outcomes using the RW approach (2–4).

Despite the promising results in RW pathways, variability in the efficacy of RW vibrolasty has been observed in clinical, human temporal bone, and *in vivo* animal studies. Early experiments with cats demonstrated potential of RW as an acoustic entry point into the cochlea by measuring cochlear potentials (5). Additionally, separate measurements of intracochlear pressure in the scala vestibuli and scala tympani in human cadavers revealed differing pressure levels across cochlear partitions when reverse stimulation was applied to the cochlea through the RW, which is comparable to applying forward stimulation to the cochlea through the OW from a normal middle ear (6, 7). Moreover, *in vivo* animal studies have shown that the auditory brainstem response to mechanical RW stimulation is comparable to that of acoustic stimulation (8). However, postoperative audiometry in clinical settings has shown inconsistent results when comparing vibroplasty via the OW and the RW (9–11). These differences may be attributed to the distinct conditions of the middle ear in patients undergoing stapes or RW vibroplasty. RW vibroplasty is often considered an alternative when OW vibroplasty is not viable. Factors such as preservation of the stapes structure and mobility impairment also affect the outcomes. However, the mechanisms and effects of these factors remain unclear. Therefore, comparing OW and RW for optimal hearing outcomes is challenging, particularly in cases involving pathological tympanic cavities.

Stapedial vibration and RW vibration have been investigated as indicators of the driving pressure of cochlear vibration because cochlear vibration is typically driven by transmitted vibration through the middle ear, and cochlear lymph is considered an incompressible fluid (12). Stapedial vibration measurement using laser Doppler vibrometry is one of the standard methods for predicting the output of AMEIs (13). Stapedial velocity when FMT-RW stimulation is applied to guinea pigs and human cadavers has been measured to evaluate cochlear input by AMEI and compare the cochlear response between RW stimulation and OW stimulation (7, 8, 14, 15). However, these studies were conducted under normal physiological conditions where stapedial motion was not impaired, limiting their applicability to most AMEI candidates with pathological middle ears. Pathological factors such as fixation of the stapes footplate or RW closure, which occurs at a high rate in chronic ear disease, could significantly affect the sound transmission efficiency from FMT to the cochlea (16). Lupo et al. (17) reported a significant increase in the threshold of cochlear microphonics compared to the normal ossicular chain when RW stimulation was applied to chinchillas with artificial stapedial fixation. However, the influence of RW fixation on the transmission efficiency of FMT vibroplasty remains unreported. Furthermore, the influence of pathological conditions of OW or RW on FMT vibroplasty in humans has not been elucidated.

The traveling wave of the basilar membrane (BM) results from pressure differences in cochlear lymph and is crucial for sound transmission within the cochlea, stimulating hair cells and generating auditory nerve responses. Studies on BM vibration through normal acoustic stimulation, known as forward stimulation, have been conducted in animal models and human cadavers (18, 19). Similarly, BM vibration via the RW has been reported in guinea pigs (20), but these studies have limitations in monitoring the entire movement of BM vibration. Moreover, comprehensive comparisons of BM vibration between OW and RW pathways based on measurements remain scarce. To determine the optimal FMT setting during VSB operation, it is crucial to understand the entire BM dynamics during intracochlear sound transmission, including OW and RW pathways. However, experimental use of the temporal bone often complicates testing due to the need to vary and systematically control multiple parameters. Furthermore, clinical data is fundamentally influenced by complex factors such as the morphology and pathology of the middle ear. This is particularly true when sound traverses the pathological middle ear in patients wearing an AMEI.

The finite element (FE) method is a sophisticated computer simulation technique that enables the modeling of complex-shaped biological structures, allowing for the quantification and visualization of their intricate movements. This method can model the inner ear under both normal and pathological conditions, facilitating the estimation and comparison of intracochlear sound transmission dynamics. Our previous studies (21, 22) evaluated changes in the vibration of the entire BM induced by pathological conditions in the cochlea, such as perilymphatic fistula and endolymphatic hydrops, using an FE model of the human cochlea. Estimations based on changes in BM vibration can evaluate sound transmission efficiency or speech recognition in VSB-implanted patients with various pathological conditions. Zhang and Gan (23) utilized an FE model, including a middle ear implantable transducer, to simulate BM vibration derived from excitation force through a transducer placed on the ossicles or the round window membrane (RWM). They found that vibration transmission efficiency was higher when applying the stimulus via the RWM using two different types of transducers. However, their study did not consider pathological changes in material properties at the stimulation site, such as stapedial fixation, nor the influence of coupling conditions. Zhao et al. (24) investigated the influence of coupling conditions in RW stimulation on intracochlear pressure using an FE model that included an electromagnetic transducer fixed to the RW niche, as suggested by Shin et al. (25) for higher transmission efficiency of RW stimulation than FMT. However, they also did not investigate the pathological influence on transfer efficiency.

This study aims to estimate the differences in postoperative hearing gain between FMT vibroplasty via the OW and RW in pathological middle ears. We investigate the dynamic behavior of the entire BM vibration using a three-dimensional FE model of the human cochlea by comparing the effects elicited by coupling the FMT to the stapes and RWM. Additionally, we estimate how sound transduction varies depending on the stiffness of the RWM or stapedial annular ligament (SAL), considering pathological fixation around the stapes footplate or RWM.

## 2 Methods

### 2.1 Finite element model of human cochlea

**Figure 1:**
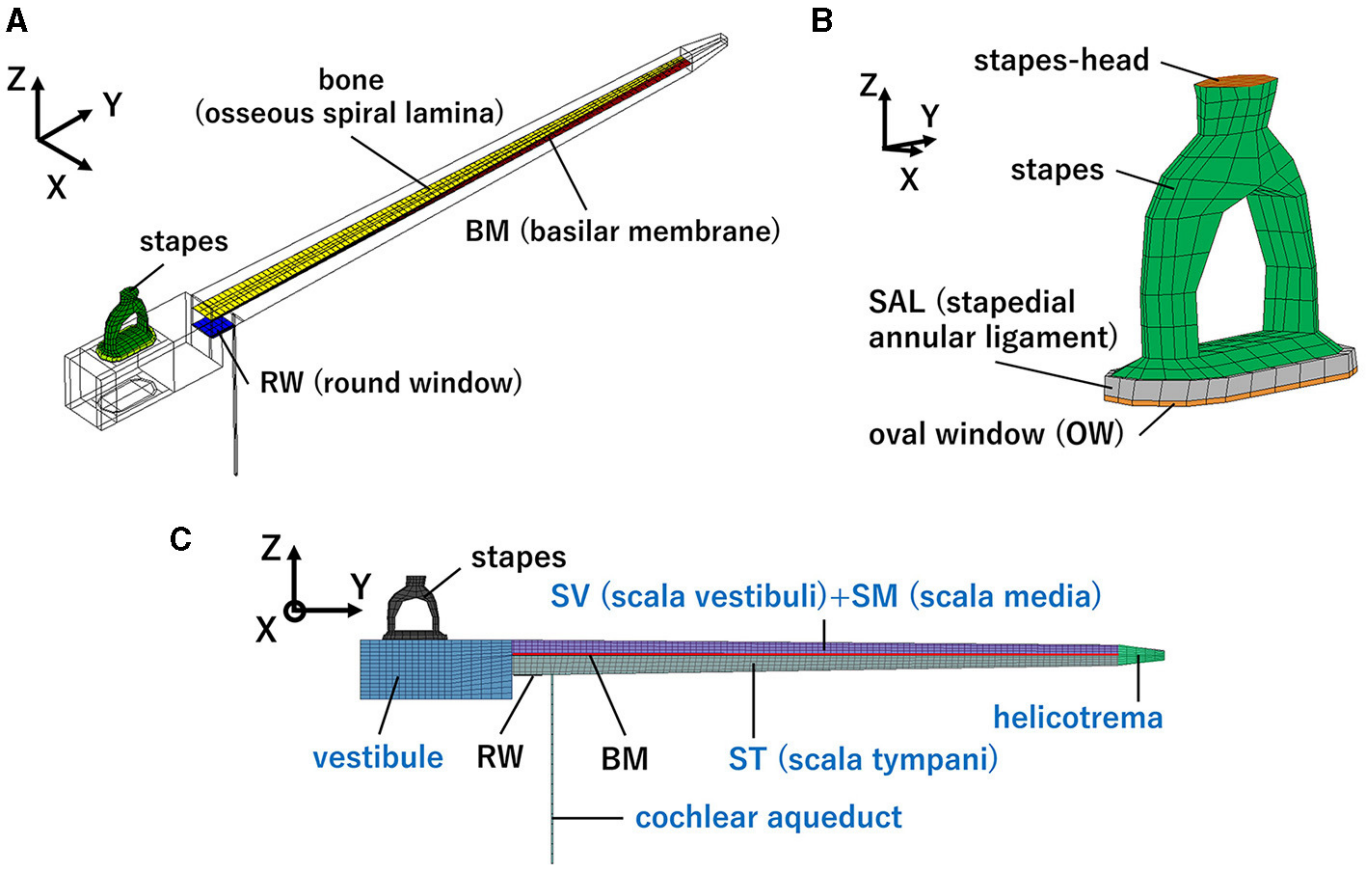
Human cochlear finite-element model: **(A)** Isometric view, **(B)** Enlarged view of the structural part around the oval window (OW), **(C)** front view. Blue letters indicate the fluid part.

The simplified straight human cochlear FE model (see Figure 1) used in this study was modified based on the model developed by Koike et al. (21). However, the geometric conditions remained unchanged. The fluid part of the cochlea (see Figure 1C) is divided into two chambers, the scala vestibuli and scala tympani, by the BM and bone (osseous spiral lamina). The two chambers are connected to a helicotrema. The scala media was considered a unified chamber with the scala vestibuli because the vibration of the BM was minimally affected (21). The material properties of the BM and RWM were varied in this study (see Table 1). The BM was assumed to be an orthotropic elastic material because the collagen fibers ran in the direction of the width of the BM (i.e., in the direction of the X-axis). Each shear modulus was calculated using the orthotropic stress-strain relations as follows in Equation 1.


(1) $$\begin{array}{c} {\text{G}}_{\text{xy}}\;=\;\frac{{\text{E}}_{\text{x}}{\text{E}}_{\text{y}}}{{\text{E}}_{\text{x}}+{\text{E}}_{\text{y}}+2{\text{E}}_{\text{y}}\nu_{\text{x}\text{y}}},\;{\text{G}}_{\text{x}\text{z}}\;=\;\frac{{\text{E}}_{\text{x}}{\text{E}}_{\text{z}}}{{\text{E}}_{\text{x}}+{\text{E}}_{\text{z}}+2{\text{E}}_{\text{z}}\nu_{\text{x}\text{z}}},\\ {\text{G}}_{\text{y}\text{z}}\;=\;\frac{{\text{E}}_{\text{y}}{\text{E}}_{\text{z}}}{{\text{E}}_{\text{y}}+{\text{E}}_{\text{z}}+2{\text{E}}_{\text{z}}\nu_{\text{y}\text{z}}} \end{array}$$

**Table 1:** Material properties of the BM and the RWM.

Basilar membrane (BM)	Young's modulus (Pa)	E_X_	2.0 × 10^6^
		E_Y_	2.0 × 10^4^
		E_Z_	2.0 × 10^6^
Round window membrane (RWM)	Young's modulus (Pa)	5.0 × 10^6^	
	Poisson's ratio	0.49	

where, *E* represents Young's modulus and *G* denotes shear modulus. Each Poisson's ratio was calculated using the orthotropic symmetry conditions as follows in Equation 2.


(2) $$\begin{array}{c} \frac{\nu_{\text{xy}}}{\nu_{\text{y}\text{x}}}=\frac{{\text{E}}_{\text{x}}}{{\text{E}}_{\text{y}}},\;\frac{\nu_{\text{x}\text{z}}}{\nu_{\text{z}\text{x}}}=\frac{{\text{E}}_{\text{x}}}{{\text{E}}_{\text{z}}},\;\frac{\nu_{\text{y}\text{z}}}{\nu_{\text{z}\text{y}}}=\frac{{\text{E}}_{\text{z}}}{{\text{E}}_{\text{y}}} \end{array}$$

where, ν represents Poisson's ratio. The other structural parts were considered isotropic elastic materials. The chambers and vestibule were filled with incompressible viscous fluid which have a density of 1, 034 kg /m^3^ and a viscosity of 0.0028 Ns /m^2^. The Rayleigh damping was set to α parameter as 100 (s^−1^), β parameter as 6.43 × 10^−7^(s) and applied to the structural parts of the model. In this study, the additional damping force, P_BM_ which is proportional to the velocity of the BM, V_BM_ is given by the formula in Equation 3.


(3) $$\begin{array}{c} P_{BM}=-c\cdot V_{BM} \end{array}$$

was applied to the surface of the BM in the direction opposite to *V*_*BM*_ in this model. The damping coefficient, *c* was set to 5,000. The peripheries of the SAL, BM, and RWM were fixed in terms of the boundary conditions. The exterior surfaces of the vestibule and chambers were assumed to be rigid walls because the cochlea is surrounded by the temporal bone. The fluid-structure interaction was considered, fluid dynamics were calculated based on the incompressible Navier–Stokes equation, and time-domain analyses were performed using CFD-ACE+ software (APPLIED MATERIALS).

### 2.2 Assessment of validity of the finite element model of human cochlea

The model was verified by comparing the experimental values of the middle ear dynamic characteristics, that is, the middle ear transfer function (METF) using the formula in Equation 4.


(4) $$\begin{array}{c} \text{METF}=20\text{lo}{\text{g}}_{10}\left(\frac{{\text{D}}_{\text{stapes}}}{{\text{P}}_{\text{EC}}}\right). \end{array}$$

Here, METF was calculated in dB ref μ*m*/*Pa* when forward stimulation was applied. *D*_*stapes*_ is the stapes footplate displacement. *P*_*EC*_ is the pressure at the ear canal, which was approximately calibrated based on an assumption of the sound pressure transmission gain in this study, as the cochlear FE model does not include the external ear canal. The sound pressure transmission gain was determined considering the surface area ratio of the tympanic membrane to the oval window membrane (OWM), and lever ratio based on the length difference between the malleal manubrium and the long process of the incus. The surface area of tympanic membrane in our FE model of human middle ear is 80 mm^2^ (26), and the surface area of OWM of the model is 5.1 mm^2^. Therefore, the gain was set to 26 dB, based on the surface ratio assumed 24 dB and the lever ratio assumed 2.5 dB (27). To validate the dynamical characteristics of the cochlea, the ratio of the volumetric displacement of RW to OW by forward stimulation was calculated and compared with the measurements. Additionally, the cochlear input impedance, *Z*_*C*_ was calculated when the forward stimulation was applied using the formula in Equation 5.


(5) $$\begin{array}{c} Z_{C}\;=\;\frac{P_{SV}}{U_{stapes}} \end{array}$$

where, *P*_*SV*_ is the pressure at the vestibule in which is 200 μ*m* from to the oval window and *U*_*stapes*_ is the volumetric velocity. Volumetric velocity was calculated by multiplying the velocities at the stapes posterior crus by the area of the stapes footplate (4.2 mm^2^). The velocities were obtained from an angle 45° with respect to the stapes footplate in the same manner of measurements by Nakajima et al. (28). Furthermore, the reverse middle ear impedance was calculated when reverse stimulation was applied to the RWM using the formula in Equation 6.


(6) $$\begin{array}{c} Z_{ME\text{\_}R}\;=\;\frac{P_{SV\text{\_}R}}{U_{stapes\text{\_}R}}. \end{array}$$

Here, *P*_*SV*_*R*_ was calculated in the same manner as *P*_*SV*_ and *U*_*stapes*_*R*_ was calculated in the same manner as *U*_*stapes*_ when the reverse stimulation was applied.

The vibration of the cochlea was calculated to simulate different sound transmission pathways. The forward stimulation and the reverse stimulation were simulated by applying a sound stimulus on the surface of the stapes head (Figure 1B) and that of RWM, respectively. The intensity of the driving force of the FMT was set to the same value, i.e., 60 dB SPL at the ear canal, regardless of the pathway to ensure a controlled and reliable comparison of the auditory responses elicited. The sound stimulus was applied as pressure on each surface and the magnitude of pressure was set to be the same force by multiplying each surface area. The surface area of the RWM of the model was 2.3 mm^2^, and that of the stapes head was 0.58 mm^2^. The sound pressure levels of the stimulus at OWM and RWM were set to 86 and 93 dB SPL, respectively. Pure-tone frequencies of 125, 250, 500, 1,000, 2,000, and 4,000 Hz were selected to verify the model because these frequencies are widely used in hearing screening.

### 2.3 Simulation of the cochlear vibration in different stimulation pathways

BM shows maximum amplitude at specific locations depending on the frequency of the sound stimulus, suggesting frequency sensitivity of the cochlea, and the frequency called characteristic frequency (CF). The auditory sensory cells on the BM are excited by the vibration amplitude of the BM where they are located (29). This suggests that CF distribution on the BM and the amplitude at CF location are related to the sensitivity of hearing. Therefore, the cochlear vibration between the stimulation methods was compared by the vibration amplitude of the BM. Changes in CF locations, where the maximum amplitude is shown by stimulation frequencies, were also compared. CF maps calculated with each excitation method were compared with the approximate curve by Greenwood (30). Here, the sound stimulus level for forward stimulation by OW vibroplasty and reverse stimulation by RW vibroplasty were set in the same way as used in the verification of the model.

An increase in the cochlear response threshold to acoustical stimulus by artificial fixation of the windows of cochlea, i.e., OW and RW, in adult fat sand rats was reported (31). It is presumed that sound delivery efficacy is weakened not only via the OW but also via the RW in these pathological conditions in the tympanic cavity, such as otosclerosis, tympanosclerosis, fibrosis, or ossification of the RWM. Additionally, several studies have reported a surgical procedure involving the placement of autograft materials between the RWM and FMT to improve vibration transmission (14, 15, 32). Placing such coupling layers could be represented by an increase in the stiffness of the RWM, considering the increment in thickness. Pathological conditions in the RWM, such as fibrosis or ossification, could also be represented by increasing the stiffness of the RWM. Impairment of stapedial mobility caused by otosclerosis or tympanosclerosis could be simulated by increasing the stiffness of SAL. The increase in Young's modulus of the SAL or the RWM was conducted in two distinct stages, i.e., 100 times magnified to their stiffness in the verified (normal) model or ossification. Ossification was represented by changing their Young's modulus to that of the bone, i.e., 200 GPa. Changes in cochlear vibration induced by the stiffness of the SAL or RWM were simulated using the two stimulation pathways. The changes in the amplitude of the BM vibration at the CF (the same frequency of the stimulus) location were calculated as changes in the hearing threshold because there is a correlation between the vibration amplitude of the BM at the CF location and the hearing threshold of that frequency. Here, the CF location of each frequency was set to the results simulated when the forward stimulation, i.e., the general sound transmission pathway, was applied to the normal model. This approach enables a comparison of changes in the hearing threshold, i.e., changes in transmission efficiency, based on the BM vibration obtained from the normal model when general forward stimulation is applied.

## 3 Results

### 3.1 Comparison between the experimental values and the simulation

**Figure 2:**
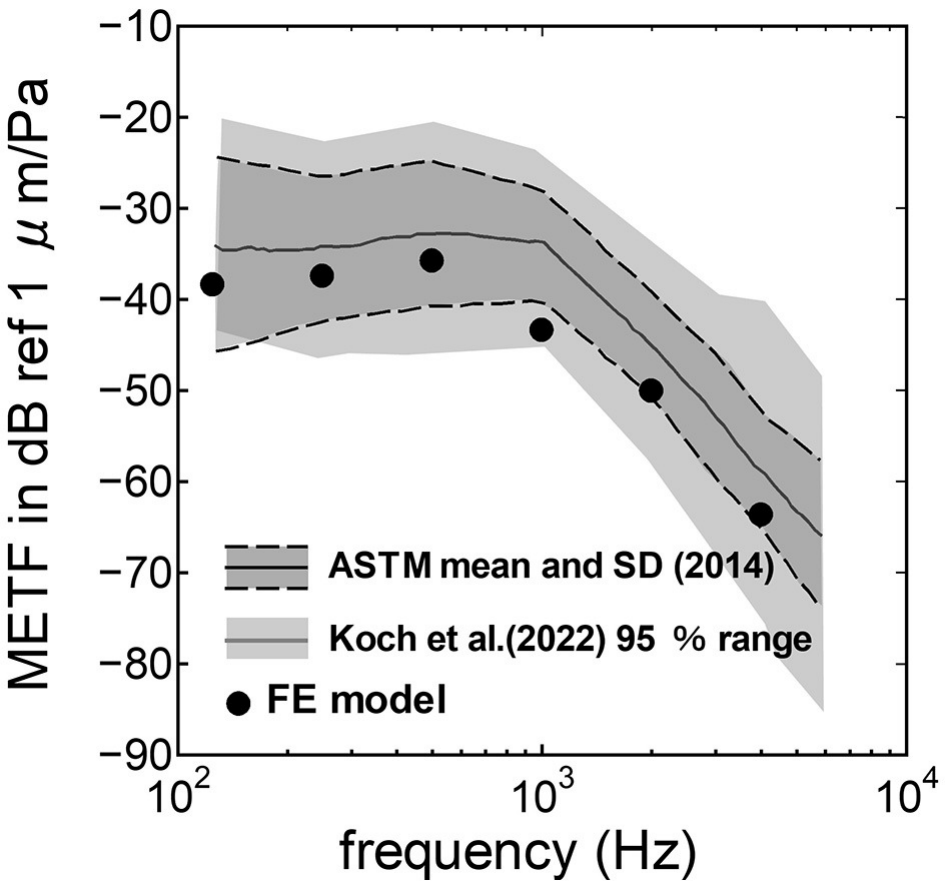
Comparison of the middle ear transfer function (METF) among the experimental values and calculation results of the FE-model. The dark gray area represents the standardized METF by ASTM (American Society for Testing and Materials) (33). The gray area indicates the 95% proportion 2-sided tolerance interval from four research groups evaluated by Koch et al. (34).

**Figure 3:**
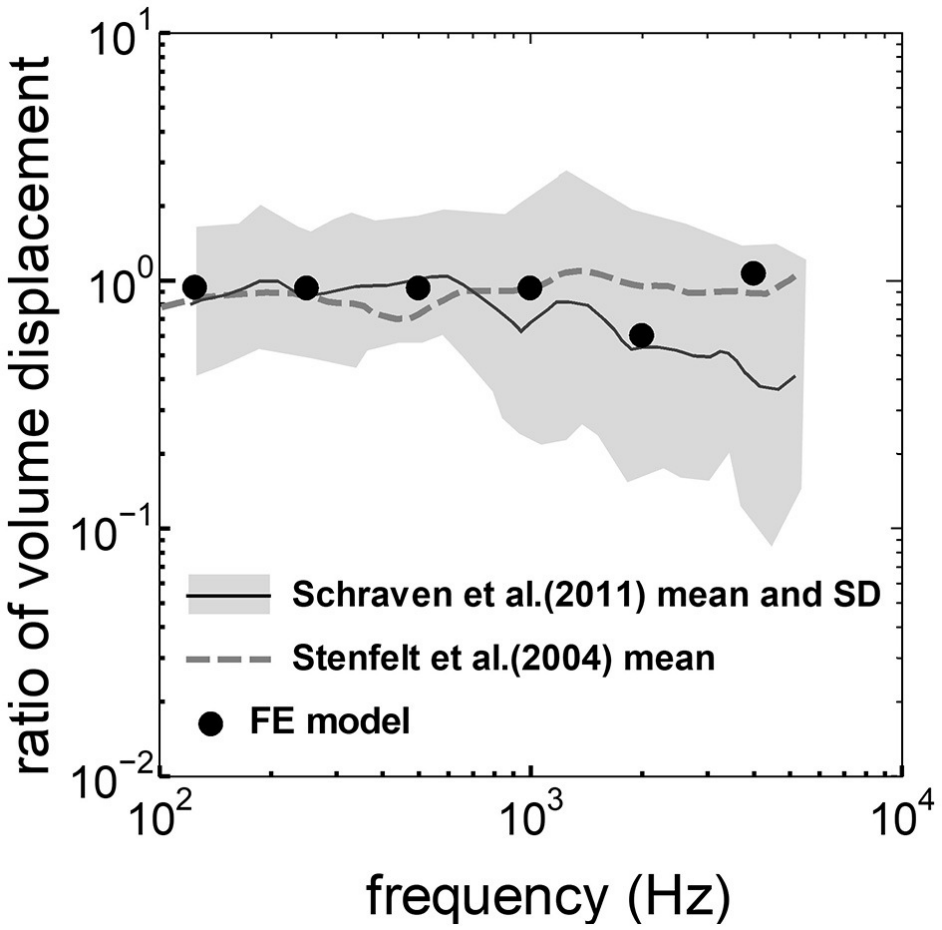
Comparison of the ratio of the volume displacement at RW to that at OW by forward stimulation among the experimental values and calculation results of the FE-model. The relative amplitude measured by Schraven et al. (35) was calibrated to the ratio of volume displacement using the mean area of RW and OW measured by Stenfelt et al. (12).

**Figure 4:**
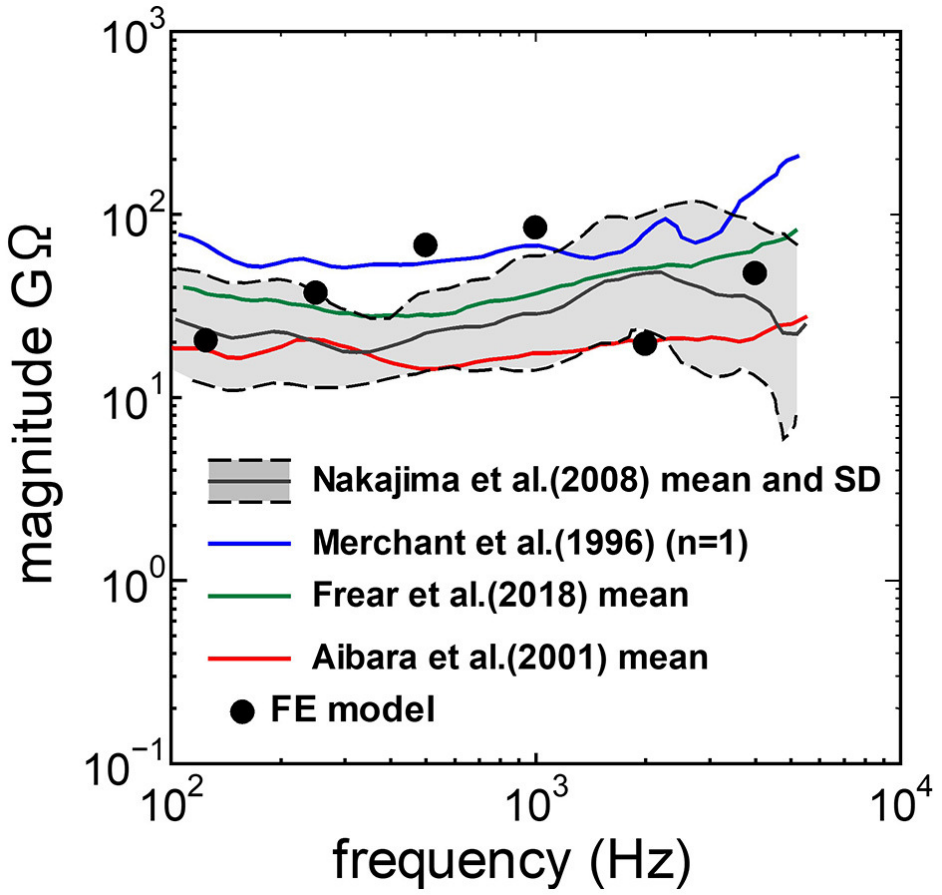
Comparison of the cochlear input impedance between the experimental values and calculation results of the FE-model.

**Figure 5:**
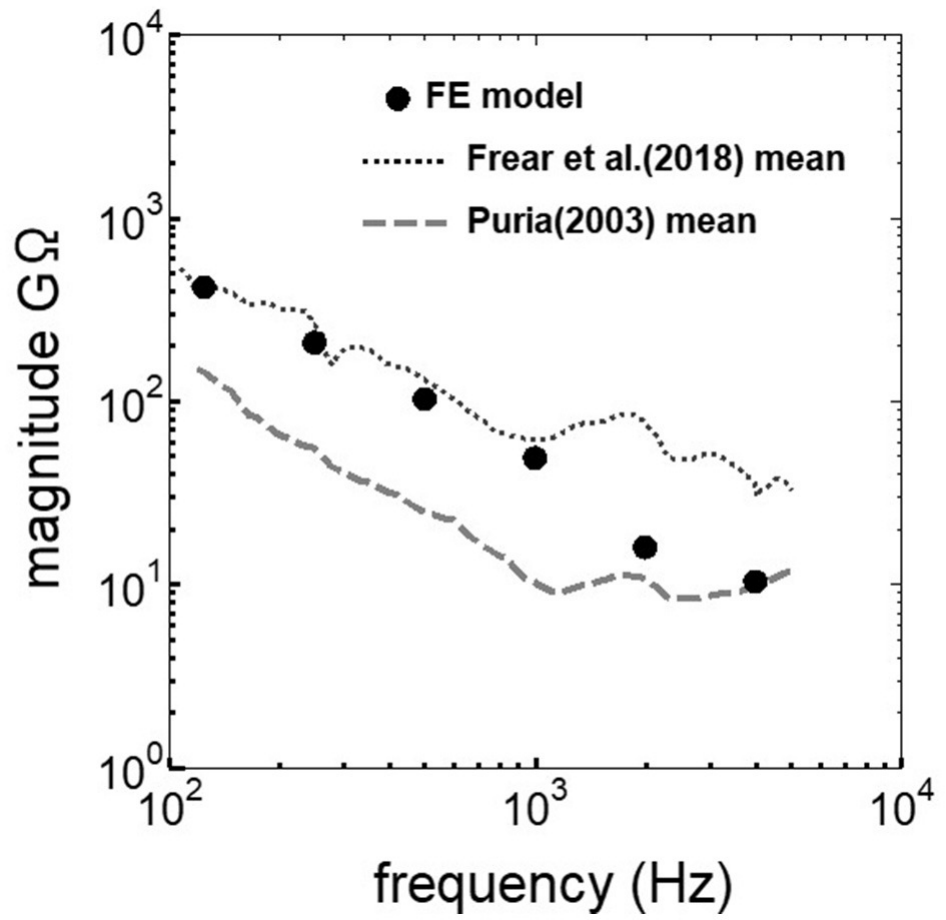
Comparison of the reverse middle ear impedance between the experimental values and calculation results of the FE-model.

The assumed METF of the FE model was compared with the “ASTM (American Society for Testing Materials) F2504_05” (33) and the experimental values (34) in Figure 2. The results obtained from the model were included in the 95% range and mean of 366 METF measurements from four different research groups (34). The ratio of volume displacement at the RW to the OW when forward stimulation was applied is shown in Figure 3. The ratio obtained from the model was matched with the experimental values in human temporal bones (12, 35). The cochlear input impedance when forward stimulation was applied (Figure 4) and the reverse middle ear impedance when reverse stimulation was applied (Figure 5) were also compared for verification. The impedances obtained from the model were mostly included in the range of experimental values from different research groups (28, 36–39).

### 3.2 Comparison of the cochlear vibration in different stimulation pathways

**Figure 6:**
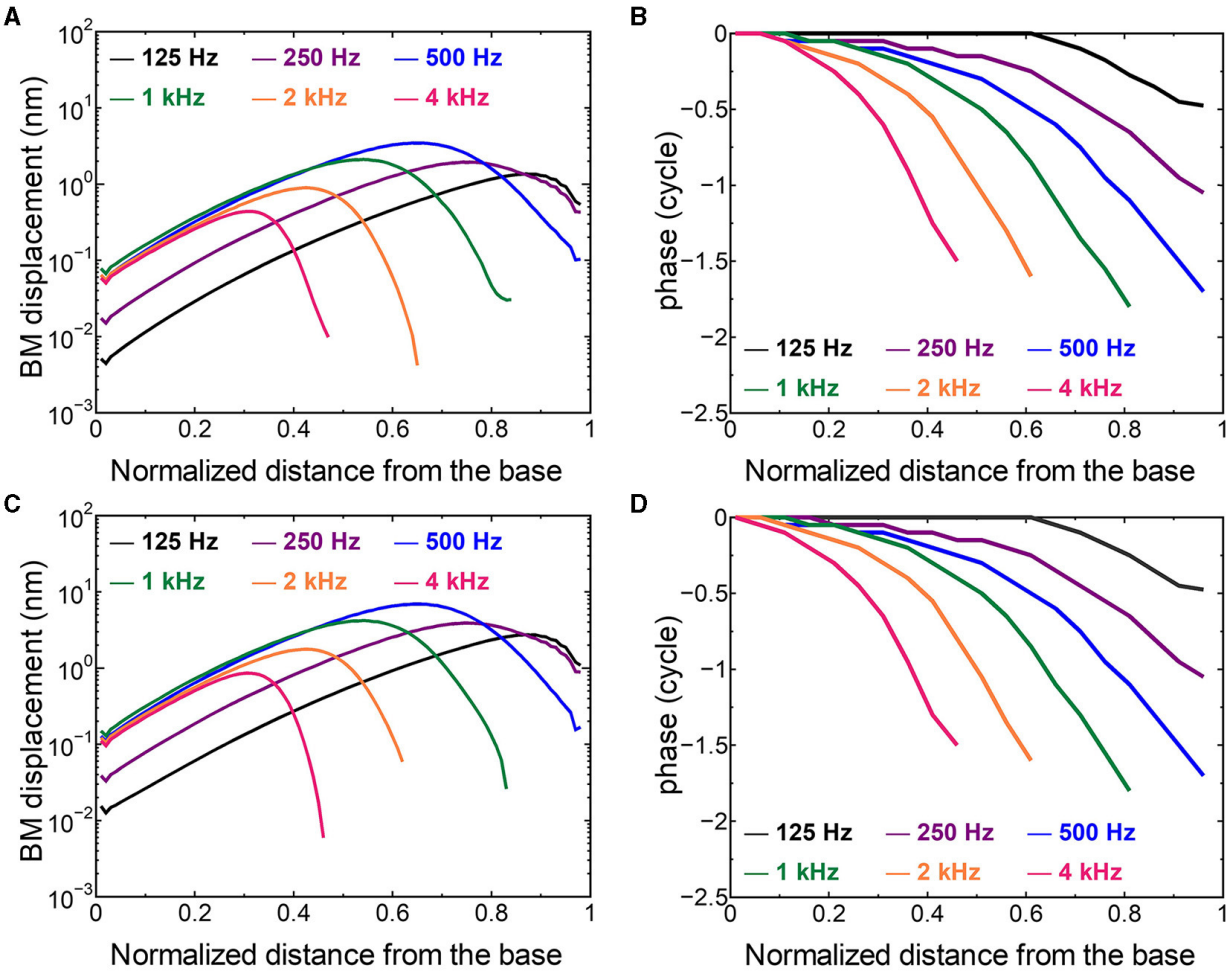
The BM displacement at frequencies of 125 Hz−4 kHz from the base to apex. **(A)** Amplitude (forward stimulus), **(B)** Phase (forward stimulus), **(C)** Amplitude (reverse stimulus), **(D)** Phase (reverse stimulus). Phase was presented in cycle re the basal edge of the BM.

**Figure 7:**
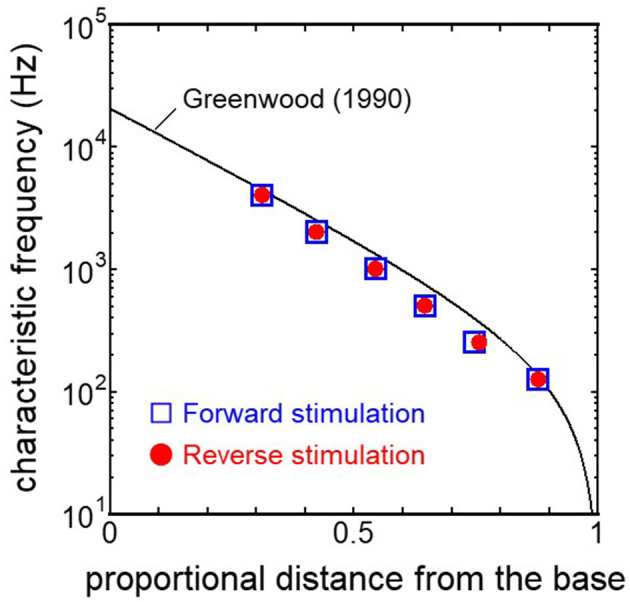
CF distribution obtained from each model. Distance from the base was normalized by BM length.

**Figure 8:**
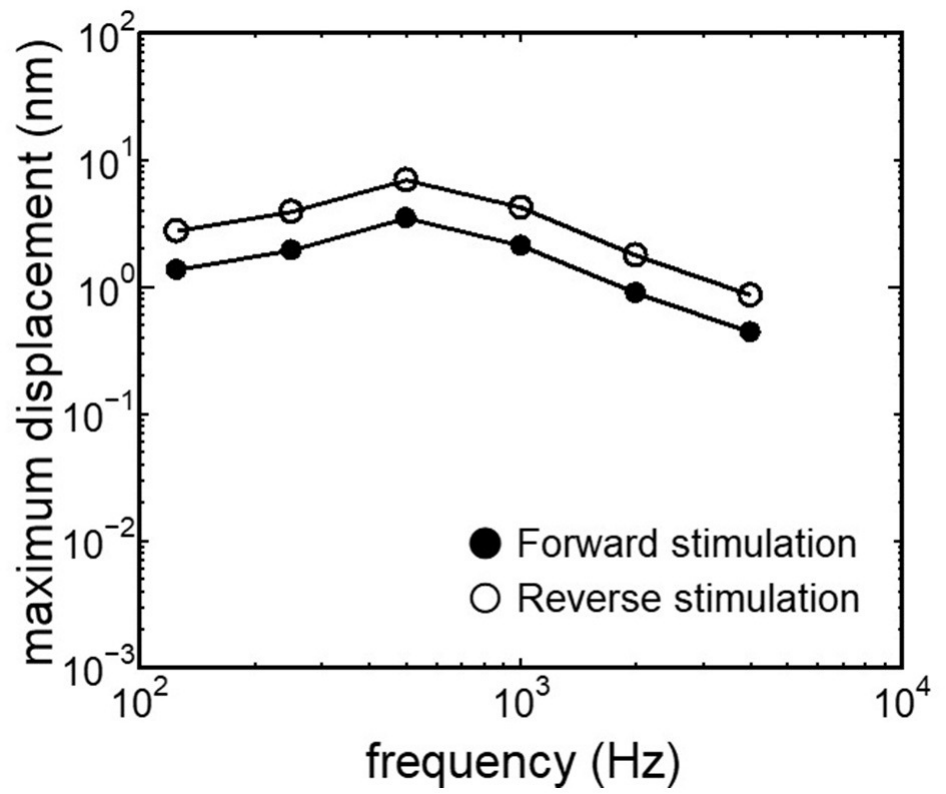
Maximum displacement of BM vibration obtained from the normal model applying forward stimulation and reverse stimulation.

The displacement and phase of the BM vibration from the base to apex were displayed in Figure 6 when forward or reverse stimulus at frequencies of 125 Hz−4 kHz was applied to the normal model. Phase was presented in cycle re the basal edge of the BM. The location of the maximum amplitude of the displacement, i.e., CF location, shifts from the base to apex with decrease of stimulus frequency. Phase at each CF location was delayed ~0.5 cycle regardless of different transmission pathways. The distribution of the CF map was consistent with the approximated curve regardless of stimulation pathways (Figure 7). CF positions were barely changed by the stimulation pathways. The maximum displacement of the BM vibration obtained from the normal model is depicted in Figure 8. The cochlear vibration derived from the reverse stimulation was larger than that derived from the forward stimulation regardless of stimulus frequency. The maximum displacement of the vibration derived from the reverse stimulation was over 5 dB larger than that derived from the forward stimulation.

**Figure 9:**
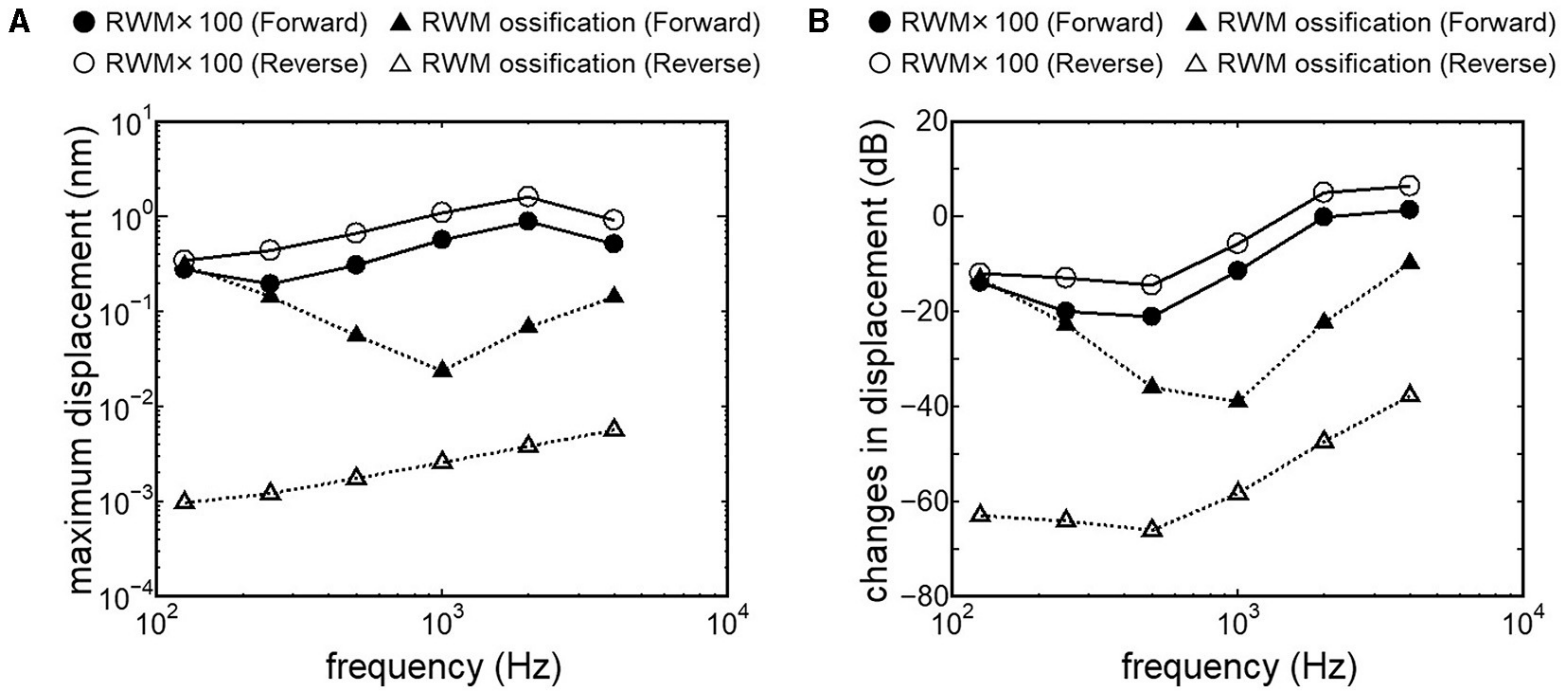
BM vibration amplitude obtained from RWM stiffened models. **(A)** Maximum displacement amplitude, **(B)** changes in vibration amplitude of RWM stiffened models to the normal model. The relative amplitudes were calculated at CF location of the normal model when the forward stimulation was applied.

**Figure 10:**
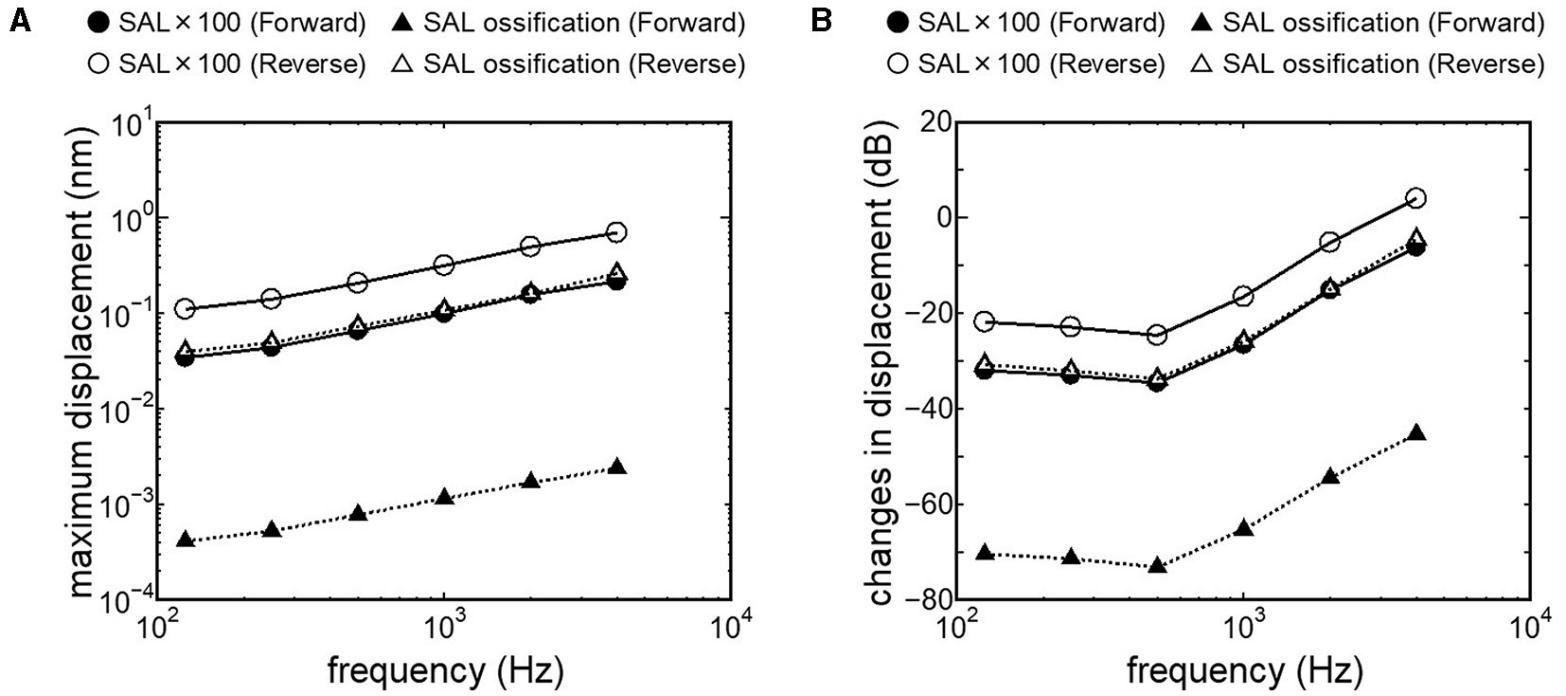
BM vibration amplitude obtained from SAL stiffened models. **(A)** Maximum displacement amplitude, **(B)** changes in vibration amplitude of SAL stiffened models to the normal model.

Results obtained from RWM-stiffened models, which represent the effect of coupling layers or pathological fixation of RWM, and SAL-stiffened models, which replicate the impairment of stapedial mobility by otosclerosis or tympanosclerosis, are shown in Figures 9, 10. Maximum displacements derived from the reverse stimulation were larger than those from the forward stimulation except for the RWM ossification model (Figures 9A, 10A). CF positions barely changed in RWM or SAL-stiffened models compared to the normal model. Changes in vibration amplitude caused by an increase in the stiffness of RWM or SAL were calculated as the relative amplitudes at the CF location of the normal model when the forward stimulation was applied (Figures 9B, 10B). “0 dB” serves as the basis for comparison, representing normal hearing by forward stimulation. The decrement of the amplitude in SAL-stiffened models was larger than that in RWM-stiffened models. The increase in the stiffness of RWM or SAL caused a large decrement of the BM vibration compared to that of the normal model with forward stimulation, especially when the low frequency of stimulus was applied (Figures 9B, 10B).

## 4 Discussion

### 4.1 Verification of validity of the finite element model of human cochlea

The human cochlear FE model was verified by comparing the FE model results with measurements in the cadaveric temporal bones (Figures 2–5). The results obtained from the FE-model were mostly included in the range of measurements by several research groups. Some of the results differed slightly from the measured results when the sound stimulus frequency was higher than 2 kHz. This is because the stapedial motion and vibrational mode shape of the RWM are affected by the stimulus frequency. Therefore, the displacements of the RWM and *U*_*stapes*_ can be varied by obtaining the points and directions. The stapes showed a piston-like motion in the frequency range lower than 2 kHz. In contrast, the stapes showed a rocking motion at frequencies higher than 2 kHz, and showed the highest rocking motion when the stimulus frequency was 2 kHz. The *U*_*stapes*_ obtained from the cadaveric studies were calculated by multiplying the stapes-footplate area by the stapedial velocity measured using laser Doppler vibrometry at one point of the stapes with specific direction which were varied among research groups and measurements. The velocity was measured in one direction, which could be influenced by stapedial motion. Therefore, *U*_*stapes*_ obtained from the FE model could differ from the measurement results when the stapes exhibited hinge movement.

The assumed METF of the FE model (Figure 2) was depicted at the lower range of measurement results. Although *P*_*EC*_ obtained from the model was simply calibrated using the surface ratio and the lever ratio regardless of the stimulus frequency, the frequency characteristic of the METF obtained from the model showed a similar tendency to the measurements. Zhang and Gan (23) simulated the vibration of the BM with a driving force of 50 μN of the transducers along the normal direction of the RWM and calibrated the equivalent sound pressure level at the ear canal of their FE model in the range from 90 to 110 dB SPL by the stimulus frequency. In this study, stimulus intensity calibrated as force by multiplying the pressure to the excitation surface area was 2.05 μN, and the sound pressure level at the ear canal, *P*_*EC*_, calibrated by simplified gain was ~60 dB SPL regardless of the frequency. A 25-fold difference in the excitation force could be calibrated as a difference in 28 dB SPL, which suggests agreements of the intensity of the stimulus and assumed *P*_*EC*_ to their simulation. This result suggests that the model could approximately represent the characteristic of the auditory periphery in the case of forward stimulation even though the middle ear part was not modeled.

The ratio of volume displacement of RWM vibration to OWM vibration was mostly a constant value around 1 in both the measurements and the simulation of the model (Figure 3). This consistency arises from the fact that the volume displacements of OW and RW should be the same, as the cochlear lymph is an incompressible fluid. This finding suggests that the FE model in the case of forward stimulation realistically represents the vibration of OWM and RWM regardless of individual anatomical differences. Validation of the FE model in different transmission pathways could be achieved by comparing the impedance in each transmission pathway (Figures 4, 5). The CF maps obtained from the model in the case of forward stimulation further validate the accuracy of the cochlear vibration. These results suggest that the normal FE model is adequate for simulating the mechanical vibration of the cochlea in different methods of vibroplasty.

### 4.2 Effectiveness of the cochlear vibration in two stimulus pathways

The CF maps obtained from the model (Figure 7) were barely influenced by the excitation methods. This result suggests that the two stimulus pathways toward the cochlea would barely affect the frequency discrimination ability. The maximum amplitude of the BM in the case of reverse stimulation was larger than that in the case of forward stimulation regardless of stimulus frequency (Figure 8). Zhang and Gan (23) also reported higher transmission efficiency in reverse stimulation than in forward stimulation. They suggested that the distances between the stimulated surface and the BM could be the reason for the difference in transmission efficiency. The ratio of stapes-footplate area (4.2 mm^2^) to RWM surface area is 1.8 in the FE model. This geometric difference could cause the difference in the amplitude of pressure to drive cochlear vibration between the stimulation pathways when the same intensity of driving force was applied. The pressure deriving cochlear vibration by reverse stimulation would be 1.8 times larger than that by forward stimulation. A doubling in pressure amplitude would correspond to an increase of ~6 dB SPL, which aligns with the relative ratio of the maximum displacement of the BM in dB (Figure 8). Therefore, the difference in pressure to drive cochlear vibration in each pathway could be a reason for the difference in vibration amplitude of the BM. According to Stenfelt et al. (12), the averaged area of the stapes-footplate was 3.85 mm^2^ and that of the RW was 2.39 mm^2^ in 15 temporal bones. The areal ratio of OW to RW could be calculated to ~1.6 in their study, and higher transmission efficiency in RW stimulation would be expected in temporal bones, although there are individual differences. However, measurement results of intracochlear pressure difference between the scala vestibule and scala tympani in human temporal bone (32, 39) and BM vibration at the basal turn of the cochlea in guinea pigs (20) reported that the forward stimulation showed higher efficiency than the reverse stimulation, especially in low frequencies under 1 kHz. They hypothesized that such low efficiency could be caused by high impedance of the middle ear in reverse stimulation or inefficient coupling of FMT to RWM.

Nakajima et al. (32) achieved optimal coupling of RWM to FMT by placing fascia between the RW and the FMT and supporting the free end of the FMT against the surrounding bone wall. They also reported that the intracochlear pressure difference with reverse stimulation for frequencies higher than 1 kHz almost reached that with forward stimulation due to a fascia-placing setting. This result is similar to the changes in BM displacement obtained from our RWM 100-fold stiffened model with reverse stimulation, depicted as open circles in Figure 9B. A 100-fold increase in stiffness could indicate a 4.6-fold increase in thickness, as stiffness is proportional to the cube of thickness. The RWM 100-fold stiffened model can be applied as a model with increased stiffness of the RW situation induced by placing fascia. Zhao et al. (24) suggested that a small Young's modulus of the coupling layer could promote coupling between the FMT and the RW based on their FE model simulation results. In cases where severe ossification is absent in the RW, and the thickening or addition of a covering layer to the RWM is minimal, it is posited that RW vibroplasty might provide superior transmission efficiency compared to OW vibroplasty.

Crompton et al. (40) reported preoperative and postoperative pure-tone hearing levels in an otosclerosis cohort (*n* = 154). The mean value of the preoperative pure-tone average (PTA) was 57 dB, and postoperatively, it was 31 dB. The increment of PTA induced by otosclerosis could be estimated by the differences between preoperative and postoperative PTA. The differences between preoperative and postoperative hearing thresholds were especially high when the frequency was lower than 1 kHz. These results are consistent with changes in BM vibration obtained from the SAL 100-fold stiffened model, depicted as closed circles in Figure 10B. Lupo et al. (17) investigated changes in cochlear thresholds induced by artificial fixation of the stapes footplate in chinchilla when RW stimulation was applied. The threshold was significantly increased by 4–13 dB, with the increment varying by frequency, showing greater increments at lower frequencies. These results align with the results obtained from the SAL 100-fold stiffened model, depicted as open circles in Figure 10B. These findings suggest that our model replicates realistic pathological conditions. The reverse stimulation showed higher maximum displacement than that derived by forward stimulation, regardless of the increment of SAL stiffness (Figure 10A). These results suggest that RW vibroplasty would have an advantage in ears with impaired stapedial mobility, such as otosclerosis or tympanosclerosis, where hearing improvement is not expected by tympanoplasty.

This novel study explored the vibration transmission efficiency of two surgical approaches for FMT placement: OW and RW vibroplasty, using an FE model of the human cochlea while considering pathological conditions in the tympanic cavity. While direct comparative analysis of hearing improvement between post-RW and -OW vibroplasty in the same human subject is impractical due to real-world clinical limitations, this approach allows for evaluating transmission efficiency without the individual anatomical and physiological variances present in actual clinical contexts. Results indicate that RW vibroplasty generally offers superior vibration transmission efficiency compared to OW vibroplasty, particularly notable in cases of stapedial mobility impairment. This finding is significant, emphasizing that RW vibroplasty typically ensures enhanced vibration transmission, barring instances of RW ossification. Additionally, transitioning to OW vibroplasty may still ensure sufficient vibratory transmission efficiency when placing FMT on the RWM is challenging due to anatomical conditions in the tympanic cavity. This is because there is only a slight difference in BM vibration between stimulus pathways, except for the ossification model. In this study, pathological conditions were represented by simply two stages of increment of stiffness. Further simulation of the FE model considering various pathological conditions and integration with clinically derived audiograms could lead to a more accurate model for discerning underlying pathologies. Lastly, extrapolating these simulated outcomes to clinical practice requires future study considering effects of stability of FMT coupling, as applying the same driving force of FMT to OWM or RWM was hypothesized in this study. Surgeons' preferences and familiarity with the coupling technique may also bias the choice of approach in vibroplasty. Furthermore, the actual hearing prognosis can be influenced by factors such as heterogeneous post-infectious sequelae of the middle ear, instability of FMT coupling, and surgical complications, including increased bone conduction thresholds.

## 5 Conclusion

The vibration transmission efficiency of two distinct surgical approaches in AMEI placement—OW vibroplasty and RW vibroplasty—was investigated using an FE model of the human cochlea. Changes in the amplitude of BM vibration in the SAL or RWM-stiffened models were evaluated as indicators of changes in hearing threshold induced by pathological conditions. Our findings revealed that RW vibroplasty generally exhibits superior vibration transmission efficiency compared to OW vibroplasty, particularly in scenarios with limited stapedial mobility. Notably, despite variations in the stiffness of RWM or SAL, RW vibroplasty maintained higher efficiency, except in cases of RW ossification. Additionally, our results suggested that transitioning to OW vibroplasty could still ensure a sufficient level of vibratory transmission efficiency when the available space within the posterior tympanic cavity is inadequate for alignment for the vertical placement of the FMT against the RWM. Moreover, our FE model can investigate changes in the transmission efficiency in various pathological conditions, and the optimal coupling method for efficient vibration transmission under RW vibroplasty. These insights not only shed light on the biomechanical underpinnings of AMEIs but also pave the way for future research aimed at optimizing vibroplasty techniques.

## Data availability statement

The original contributions presented in the study are included in the article/supplementary material, further inquiries can be directed to the corresponding author.

## Author contributions

SL: Data curation, Formal analysis, Funding acquisition, Investigation, Methodology, Project administration, Software, Validation, Visualization, Writing – original draft, Writing – review & editing, Conceptualization. MM: Conceptualization, Investigation, Project administration, Writing – review & editing, Writing – original draft. TK: Methodology, Project administration, Supervision, Writing – review & editing.
